# Parenting stress, anxiety, and sources of acquiring knowledge in Taiwanese caregivers of children with attention-deficit/hyperactivity disorder

**DOI:** 10.1186/s12889-024-18761-x

**Published:** 2024-06-24

**Authors:** Tai-Ling Liu, Ray C. Hsiao, Wen-Jiun Chou, Cheng-Fang Yen

**Affiliations:** 1 Department of Psychiatry, School of Medicine, College of Medicine, Kaohsiung Medical University, and Department of Psychiatry, Kaohsiung Medical University Hospital, 100 Tzyou 1st Road, Kaohsiung, 80708 Taiwan; 2 Department of Psychiatry, Seattle Children’s, Seattle, and Department of Psychiatry and Behavioral Sciences, School of Medicine, University of Washington, Seattle, WA USA; 3 Department of Child and Adolescent Psychiatry, Chang Gung Memorial Hospital, Kaohsiung Medical Center, 32 Dapi Rd. Niaosong Dist, Kaohsiung, 83341 Taiwan; 4 School of Medicine, Chang Gung University, Taoyuan, Taiwan; 5 Department of Psychiatry, Kaohsiung Medical University Hospital, and School of Medicine College of Medicine, Kaohsiung Medical University, 100 Tzyou 1st Road, Kaohsiung, 80708 Taiwan; 6 College of Professional Studies, National Pingtung University of Science and Technology, Pingtung, Taiwan

**Keywords:** Anxiety, Attention-deficit/hyperactivity disorder, Caregiver, Parenting stress, Social media, Psychological well-being

## Abstract

**Background:**

This survey study investigated the types of sources other than medical professionals (e.g., social media) that the caregivers of children with attention-deficit/hyperactivity disorder (ADHD) use to acquire knowledge about ADHD and investigated the association between the use of such information sources and caregiver parenting stress and anxiety in Taiwan.

**Methods:**

A total of 213 caregivers of children with ADHD participated in this study. The sources that the caregivers used to acquire knowledge about ADHD other than medical professionals were investigated. Caregiver parenting stress was assessed using the Parenting Stress Index, and caregiver anxiety was assessed using the Beck Anxiety Inventory. The associations of the types of sources used and total number of source use with caregiver parenting stress and anxiety were investigated using multivariate linear regression analysis.

**Results:**

The most common source of knowledge other than medical professionals was teachers (55.4%), followed by social media (52.6%), traditional media (50.7%), friends (33.8%), caregivers of other children (21.1%), and family members (18.3%). The caregivers’ mean total number of using sources of knowledge about ADHD other than medical professionals was 2.32. Acquiring knowledge about ADHD from social media was significantly associated with caregiver parenting stress. Additionally, acquiring knowledge about ADHD from caregivers of other children was significantly associated with caregiver parenting stress and anxiety, as was the frequency of using sources of knowledge about ADHD other than medical professionals.

**Conclusion:**

The caregivers of children with ADHD acquired knowledge about ADHD from multiple sources. Acquiring knowledge about ADHD from social media was significantly associated with caregiver parenting stress. The number of sources of knowledge about ADHD was significantly associated with caregiver parenting stress and anxiety.

## Background

Attention-deficit/hyperactivity disorder (ADHD) is the most common neurodevelopmental disorder in the world [1]; a systematic review and meta-analysis indicated a global prevalence of 7.2% [2]. The symptoms of ADHD not only result in multiple functional impairments in individuals with ADHD [3] but also exacerbate parenting stress and mental health conditions among caregivers [4, 5]. Studies have determined that caregiver mental health conditions were significantly associated with the exacerbation or persistence of child ADHD symptoms [6] and poor caregiver–child relationships [7]. Helping caregivers improve their ability to cope with their child’s ADHD is thus crucial to the well-being of both children and caregivers.

The national health insurance system enhances the availability of mental health care for children and adolescents in Taiwan. However, ADHD is underdiagnosed and undertreated in Taiwan [8]. One of the reasons is the public’s stigmatizing attitude toward children with ADHD and their caregivers in Taiwan [Chang 2020]. Caregivers with affiliate stigma have unfavorable attitudes toward children’s diagnoses, pharmacotherapy and behavioral therapy, and etiological explanations for ADHD [9]; caregivers with affiliate stigma tend to employ complementary and alternative intervention strategies for their children’s ADHD [10]. Moreover, some people in Taiwan urge caregivers not to allow their children to receive psychiatric care because of religious beliefs and opposition to biological etiologies of ADHD. Therefore, helping caregivers obtain accurate knowledge of ADHD is important for getting early help for their children.

According to the health belief model [11], knowledge about ADHD is essential to motivating caregivers to work with health-care professionals to improve children’s ADHD through medication and psychotherapy. One study indicated that caregiver-reported knowledge about ADHD was associated with a greater likelihood of enrolling their child in treatment [12], whereas another study revealed that caregiver knowledge about ADHD gained during treatment reduced caregiver psychological distress [13]. Improving caregiver knowledge about ADHD is therefore a critical task for health-care professionals.

Caregivers often acquire knowledge about ADHD from health-care professionals during office visits [14–16], but they may also acquire information from a wide range of other sources [15, 16]. The Internet is frequently a primary source of information for caregivers seeking information about ADHD [15–17]. Internet searches play a key role in increasing awareness of child ADHD [18] because familiarity with Internet-based information sources prompts many caregivers to use the Internet to find information about their children’s medical conditions [19]. However, information about ADHD obtained from the Internet is often of low quality, unreliable, inaccurate, and at odds with evidence-based practices [20, 21], and therefore, caregivers are vulnerable to receiving misinformation that may delay their child’s treatment [22]. A study reported that misconceptions about ADHD were associated with lower acceptance of medication and greater acceptance of nonpharmacological dietary interventions [21].

Social media platforms are a popular means of sharing medical information online. These platforms use proprietary algorithms to increase user engagement and may promote sources that do not present accurate health information [23]. A systematic review revealed that the prevalence of health misinformation was high across the majority of social media platforms [24]. Nevertheless, the associations between the acquisition of knowledge about ADHD from social media, parenting stress, and mental health conditions remains poorly understood. Given the significant impact of social media messaging on modern life, it is important to investigate the association between caregivers’ acquiring knowledge of ADHD from social media and parenting stress.

Social media platforms are not the only sources of knowledge about ADHD other than medical professionals that are available to caregivers. Teachers, traditional media, caregivers of other children, family members, and friends can serve as sources of information about ADHD. Several studies in Asian countries have examined the knowledge of ADHD and attitudes toward the individuals with ADHD among community members. For example, a study in Indonesia found that between 52.1% and 65.6% of community members, primary school teachers, medical students, general practitioners, pediatricians and psychologists have poor to very poor levels of knowledge of ADHD [25]. A study in Korea revealed that only 23.9% of community members can recognize ADHD symptoms described in a vignette correctly [26]. A study in India found that 56% of primary school teachers did not have any knowledge regarding ADHD [27]. The results of previous studies indicate that caregivers of children with ADHD may receive incorrect knowledge about ADHD from the people around that may affect their interactions with their children. The effects of obtaining knowledge from these sources on parenting stress and mental health conditions have not been well studied.

The current study analyzed the types of sources beyond medical professionals that the caregivers of children with ADHD frequently used to acquire knowledge about ADHD and the association between source use and caregiver stress and anxiety. Because of the high prevalence of health misinformation on social media [24] and the potential of misinformation to delay treatment [21], we hypothesized that acquiring knowledge about ADHD from social media would be significantly associated with parenting stress and anxiety among the caregivers of children with ADHD. Because caregivers may acquire evidence-based knowledge about ADHD from either children’s teachers or health-care professionals (in the form of traditional media) but may receive inaccurate information from the caregivers of other children, family members, or friends, we further hypothesized that different sources of knowledge about ADHD would have different associations with parenting stress and anxiety.

## Methods

### Participants and procedure

This study recruited participants from three child and adolescent psychiatric clinics of two general hospitals in southern Taiwan. Participants were required to be primary caregivers of a child aged 10 to 18 years with a diagnosis of ADHD based on the criteria of the *Diagnostic and Statistical Manual of Mental Disorders, Fifth Edition (DSM-5)* [1]. Three child psychiatrists conducted clinically diagnostic interviews with the children and caregivers and made the DSM-5ADHD diagnosis based on children’s self-reports, caregivers’ observation, teachers’ observation relayed by caregivers, and psychiatrists’ observation for children’s behaviors in the clinics. Children who had an intellectual disability or autism spectrum disorder with communication difficulty were excluded. Main family caregivers meant family caregivers who spent the most time on caring for the children with ADHD compared with other caregivers. Main caregivers who had an intellectual disability, schizophrenia, bipolar disorder, or any cognitive deficits that resulted in significant difficulties in communication were also excluded. Three child psychiatrists had excellent interrater agreement, and the kappa value for the diagnosis of ADHD was 1 among the six case videos before the formal conduction of this study. Psychiatrists ascertained that the children had no diagnoses of intellectual disability, autism spectrum disorder, bipolar spectrum disorder, schizophrenia spectrum disorder, or psychotic disorder based on the interviews with caregivers and medical chart records. Psychiatrists also excluded caregivers who had the diagnoses of schizophrenia spectrum disorder, intellectual disability, substance use disorder, or other physical or psychiatric disorder that could impair their ability to understand the aims and procedures of the study based on the results of interviews with caregivers.

Caregivers of children with ADHD who visited the outpatient clinics of the two hospitals between June 2018 and May 2021 were consecutively invited to participate in the study. Three child psychiatrists initially identified 220 caregivers who were eligible to participate; of these caregivers, 213 (168 women and 45 men) agreed to participate and provided informed consent. Research assistants explained how the self-report questionnaires should be completed; the participants then individually completed the questionnaires in research rooms. This study was approved by the institutional review boards of the two participating hospitals (201800740A3 and KMUHIRB-SV(II)-20,170,077).

### Measures

#### Sources of knowledge about ADHD

We asked the caregivers how frequently they used the following six sources to acquire knowledge about ADHD: social media, including Facebook, LINE, Instagram, and Twitter; teachers; traditional media, including television and newspapers; caregivers of other children; family; and friends. Facebook, Instagram and Twitter are the most popular social media among Taiwanese people in 2018. LINE is the most popular communication media in Taiwan; LINE’s various social networking features also make it a complete social media package. Potential responses were *never*, *seldom*, *sometimes*, and *frequently*. Caregivers who responded *sometimes* or *frequently* were classified as having used a source to acquire knowledge about ADHD. After the surveys were collected, the sources the caregivers used were summarized and used in the next stage of analysis.

#### Parent form of the swanson, nolan, and pelham scale, version IV

We asked the caregivers to rate the severity of their child’s ADHD and oppositional defiant disorder symptoms in the previous 30 days by using the Chinese version [28] of the Parent Form of the Swanson, Nolan, and Pelham Scale, Version IV (PF-SNAP-IV) [29]. The 26 items on the PF-SNAP-IV are categorized under the 3 subscales of inattention, hyperactivity/impulsivity, and oppositional defiance. The participants rated each item on a 4-point scale with endpoints ranging from 0 (*not at all*) to 3 (*very much*). A higher total subscale score indicated higher levels of inattention, hyperactivity/impulsivity, and symptoms of oppositional defiant disorder, with the Cronbach’s α values in this study for the three subscales being 0.89, 0.90, and 0.93, respectively.

#### Parenting stress index, fourth edition short form

We used the Taiwanese version [30] of the Parenting Stress Index, Fourth Edition Short Form (PSI-4-SF) [31] to assess caregiver-reported parenting stress. The participants rated each item on a 5-point scale with endpoints ranging from 1 (*strongly disagree*) to 5 (*strongly agree*). A higher total subscale score indicated higher parenting stress. The three subscales of the original PSI-4-SF were reported to have good to satisfactory internal consistency (Cronbach’s α: 0.88 to 0.90) and acceptable to good test–retest reliability (test–retest coefficient: 0.68 to 0.85) [31]. The Taiwanese version of the PSI-4-SF was also reported to have good to satisfactory internal consistency (Cronbach’s α: 0.86 to 0.91) [30]. The Cronbach’s α for the PSI-4-SF in this study was 0.90.

### Beck anxiety inventory

We used the 21-item Chinese version [32] of the Beck Anxiety Inventory (BAI) [33] to assess caregiver-reported anxiety. The participants rated each item on a 4-point scale with endpoints ranging from 0 (*not at all*) to 3 (*severely*). A higher total score indicated higher anxiety. The original version of the BAI was reported to have good to satisfactory internal consistency (Cronbach’s α: 0.85 to 0.93), acceptable test–retest reliability (test–retest coefficient: 0.75), and acceptable convergent validity with the Hamilton Anxiety Rating Scale (HARS; Pearson’s correlation coefficient: 0.51) [33]. The Chinese version of the BAI was reported to have good internal consistency (Cronbach’s α: 0.95) and acceptable convergent validity with the HARS (Pearson’s correlation coefficient: 0.72) [32]. The Cronbach’s α for the BAI in this study was 0.94.

### Demographic characteristics

We collected data on the caregivers’ sex, age, and years of education completed. We also collected data on their children’s sex and age.

### Statistical analysis

All statistical analyses were conducted using SPSS 24.0 (SPSS, Chicago, IL, USA). Continuous variables are presented as means and standard deviations (SDs), whereas categorical variables are presented as percentages. Multivariate linear regression was used to analyze the associations between sources of knowledge about ADHD and caregiver parenting stress and anxiety, with caregiver and child demographics and child inattention, hyperactivity/impulsivity, and oppositional defiance included as covariates. We considered a *p* value of < 0.05 to be statistically significant.

## Results

Table 1 presents the demographic characteristics of the 213 caregivers. The caregivers were grouped by sex (168 women and 45 men), mean (SD) age in years (44.63 [6.11]), and mean [SD] level of education in years (14.15 [3.02]). The children with ADHD were grouped by sex (32 girls and 181 boys) and mean (SD) age in years (12.88 [2.15]). The mean (SD) values for inattention, hyperactivity/impulsivity, and oppositional defiance measured using the PF-SNAP-IV were 13.18 (5.90), 9.01 (5.89), and 9.57 (6.03), respectively. The mean (SD) value for parenting stress on the PSI-4-SF was 97.28 (23.91). The absolute skewness and kurtosis values for the PSI-4-SF scores were 0.102 and 0.157, respectively. The mean (SD) value for caregiver anxiety on the BAI was 8.07 (9.43). The absolute skewness and kurtosis values for the BAI scores were 1.952 and 5.003, respectively. On the basis of Kim’s article [34], we concluded that the scores for parenting stress and anxiety in this study were normally distributed.

**Table 1:** Caregiver and child factors and parenting stress (*N* = 213)

	*n* (%)	Mean (SD)	Range
*Caregiver factors*			
Sex			
Male	45 (21.1)		
Female	168 (78.9)		
Age (years)		44.63 (6.11)	30–69
Years of education (years)		14.15 (3.02)	6–24
Parenting stress on the PSI		97.28 (23.91)	36–166
Anxiety on the BAI		8.07 (9.43)	0–54
*Child factors*			
Sex			
Boy	181 (85.0)		
Girl	32 (15.0)		
Age (years)		12.88 (2.15)	10–18
Inattention		13.18 (5.90)	1–27
Hyperactivity/impulsivity		9.01 (5.89)	0–26
Oppositional defiance		9.57 (6.03)	0–24

BAI: Beck Anxiety Inventory; PSI: Parenting Stress Index

Table 2 presents the percentages of caregivers acquiring knowledge about ADHD from various sources. The most frequently used source of information was teachers (55.4%), followed by social media (52.6%), traditional media (50.7%), friends (33.8%), caregivers of other children (21.1%), and family members (18.3%). The mean (SD) total number of sources of knowledge about ADHD was 2.32 (1.52).

**Table 2:** Sources of knowledge about ADHD used by caregivers (*N* = 213)

	*n* (%)
Social media	112 (52.6)
Children’s school teachers	118 (55.4)
Traditional media	108 (50.7)
Friends	72 (33.8)
Parents of other children	45 (21.1)
Families	39 (18.3)
Number of knowledge sources	
0	24 (11.3)
1	49 (23.0)
2	49 (23.0)
3	42 (19.7)
4	27 (12.7)
5	19 (8.9)
6	3 (1.4)

Table 3 presents the results of linear regression analysis of the association between the use of knowledge sources about ADHD and caregiver parenting stress and anxiety. The results indicated that children’s inattention and ODD symptoms and acquiring knowledge about ADHD from social media was significantly associated with caregiver parenting stress. Acquiring knowledge about ADHD from the caregivers of other children was also significantly associated with caregiver parenting stress and anxiety.

**Table 3:** Associations between caregiver and child factors and sources of knowledge about ADHD with respect to caregiver parenting stress and anxiety: multivariate linear regression

	Parenting stress	Anxiety
	B (se)	B (se)
Caregivers’ sex	0.924 (3.644)	-1.809 (1.590)
Caregiver age	0.187 (0.261)	-0.085 (0.114)
Caregiver education	-0.305 (0.496)	-0.058 (0.216)
Children’s sex	-1.270 (4.120)	0.730 (1.798)
Children’s age	0.679 (0.744)	0.353 (0.325)
Children’s inattention	0.937 (0.302)**	-0.011 (0.132)
Children’s hyperactivity/impulsivity	-0.219 (0.353)	0.118 (0.154)
Children’s oppositional defiance	1.078 (0.321)**	0.247 (0.140)
Children’s school teachers	-3.496 (3.031)	-0.263 (1.323)
Social media	9.066 (3.392)**	-0.730 (1.480)
Traditional media	-2.728 (3.179)	-0.202 (1.387)
Friends	0.629 (3.450)	1.485 (1.505)
Parents of other children	9.214 (4.020)*	4.572 (1.754)*
Family	4.721 (3.867)	1.292 (1.687)

*p < 0.05, **p < 0.01

Table 4 presents the results of the linear regression analysis of the association between the total number of the aforementioned sources of knowledge about ADHD and caregiver parenting stress and anxiety. The results indicated that the number of sources of knowledge about ADHD was significantly associated with caregiver parenting stress and anxiety.

**Table 4:** Associations between caregiver and child factors and frequency of using sources of knowledge about ADHD with respect to caregiver parenting stress and anxiety: multivariate linear regression

	Parenting stress	Anxiety
	B (se)	B (se)
Caregivers’ sex	1.057 (3.666)	-1.672 (1.568)
Caregiver age	0.252 (0.258)	-0.119 (0.110)
Caregiver education	-0.231 (0.503)	-0.069 (0.215)
Children’s sex	-0.049 (4.200)	0.740 (1.796)
Children’s age	0.221 (0.748)	0.339 (0.320)
Children’s inattention	0.888 (0.308)**	-0.017 (0.132)
Children’s hyperactivity/impulsivity	-0.185 (0.359)	0.144 (0.154)
Children’s oppositional defiance	1.243 (0.322)***	0.265 (0.138)
Number of knowledge sources	2.520 (0.994)*	0.858 (0.425)*

*p < 0.05; **p < 0.01; ***p < 0.001

## Discussion

The present study discovered that social media, teachers, and traditional media were the three main sources of knowledge used by Taiwanese caregivers of children with ADHD. Acquiring knowledge about ADHD from social media was significantly associated with caregiver parenting stress. Additionally, acquiring knowledge about ADHD from the caregivers of other children was significantly associated with caregiver parenting stress and anxiety, as was the total number of sources for acquiring knowledge about ADHD other than medical professionals.

Several mechanisms may account for the significant association between acquiring knowledge about ADHD from social media and caregiver parenting stress. First, the knowledge about ADHD that spread in social media is highly reflective of the attitudes of the people in this society towards ADHD. Public stigma toward ADHD is common in Taiwan [9]. Taiwanese people are affected by the collectivism and have a low tolerance to behaviors that interferes with harmony. Children with ADHD symptoms such as hyperactivity and impulsivity are often viewed by the public as the spoilers of harmony. The public often attribute these ADHD to the caregivers’ failure in discipline. These public attitudes toward ADHD spread in social media may exacerbate caregiver parenting stress. Moreover, in Taiwan, some self-proclaimed human rights groups promote the notion in social media that allowing children to receive ADHD medication is detrimental to their health. Caregivers who receive these messages from social media are often hesitant to allow their children to undergo psychiatric evaluations and treatments; caregiver parenting stress increases consequently. Second, some of the members of social media groups are relatives or friends that caregivers know well. Caregivers may perceive the stress associated with the relationship when receiving knowledge about ADHD shared by these friends and family members. Third, studies have reported that a high proportion of information regarding ADHD shared on social media platforms, such as YouTube [35] and TikTok [23], is misleading. Caregivers may therefore acquire inaccurate knowledge regarding the symptoms, etiologies, and treatment of ADHD from social media and use it to make poor decisions about whether to seek treatment. These poor decisions may cause caregivers to not seek treatment for their child’s ADHD, increasing the challenge of parenting and therefore caregiver stress. Fourth, caregivers who already had high parenting stress may have been more inclined to seek parenting advice or emotional support from others, including people on social media. Fifth, both high parenting stress and overuse of social media may be symptoms of caregiver mental health conditions [36] or coping mechanisms being ineffective in managing the challenges posed by their child’s ADHD [37].

These findings indicate that the public may benefit if health-care professionals upload high-quality videos and messages to social media that provide accurate information about ADHD. Correct knowledge of ADHD is positively associated decreased social distance toward persons with ADHD [26]; thus, the stress experienced by the caregivers of children with ADHD may decrease. In addition, the caregivers of children with ADHD must exercise caution in distinguishing between inaccurate and accurate information regarding ADHD. Furthermore, the present study examined whether caregivers used Facebook, LINE, Instagram, and Twitter to acquire knowledge about ADHD; in light of the rise of various social media such as TikTok, the influence of ADHD knowledge acquired by caregivers from these emerging social media on parenting stress and anxiety warrants further study.

The present study also discovered that acquiring knowledge about ADHD from the caregivers of other children was significantly associated with caregiver parenting stress and anxiety. One explanation for this association is that the knowledge obtained from other caregivers was not so accurate compared with that obtained from health professionals. Caregivers of other children may deliver the unverified and unscreened knowledge about ADHD acquired from various sources to caregivers of children with ADHD. Moreover, studies have found that individuals who have practice experiences in interacting with children with ADHD have a higher level of knowledge, positive perception and non-stigmatizing attitude towards ADHD compared with those who have no practice experiences [25, 26]. Therefore, the knowledge obtained from other caregivers may not reflect the unique situation of the children with ADHD and the caregivers for whom it was intended, which increased stress by causing the caregivers to wrongly suppose that the information about ADHD available to them was unhelpful and unreliable. Caregivers may also have experienced stress when the caregivers of other children combined advice with criticism of a child’s ADHD symptoms or disruptive behaviors. Alternatively, caregivers who already experienced high parenting stress and anxiety may have been more inclined to seek the advice of the caregivers of other children. These possibilities further highlight the need to provide the general public with accurate and reliable information about ADHD.

Although we hypothesized that caregivers of children with ADHD were likely to seek information about their children’s condition from teachers, this study did not reveal a significant association between caregivers acquiring knowledge about ADHD from teachers or traditional media and parenting stress and anxiety. Moreover, this study analyzed the frequency of acquiring knowledge about ADHD from various sources but not the contents of those sources. For these reasons, we were unable to ascertain whether school teachers and traditional media provided more accurate knowledge about ADHD than other sources did or whether caregivers fully understood the knowledge they acquired. However, because this study discovered that teachers were the most common source of knowledge about ADHD for caregivers, the public may benefit from information regarding how to educate caregivers about ADHD being incorporated into teacher training. Additionally, the government and health-care professionals can benefit the public by providing reliable, accurate knowledge about ADHD through traditional media.

The present study identified positive associations between the total number of sources used for acquiring knowledge about ADHD and caregiver parenting stress and anxiety. One explanation for this finding is that knowledge about ADHD from different sources was often contradictory and confusing for caregivers. Alternatively, caregivers with high parenting stress and anxiety may have sought knowledge about ADHD from multiple sources to address difficulties encountered in caregiving. Because access to reliable information can help caregivers cope with their child’s condition and regain a sense of control over their caregiving [38], caregivers must be trained in appraising the authenticity of the information they obtain and its applicability to their child’s situation.

In addition to the sources to acquire knowledge about ADHD, the present study found that children’s inattention and ODD symptoms positively related to caregiver parenting stress. Caregivers repeatedly remind their children with ADHD of the things they should pay attention to in their daily lives. Especially, the education system in Taiwan is strongly influenced by ideas in Confucianism, which emphasizes hard work, effort, persistence, self-cultivation, and discipline in one’s studies [39]. The core symptoms of ADHD adversely affect a child’s ability to perform academically. Consequently, caregivers experience great parenting stress when they monitor their children’s learning. Previous studies have revealed that more severe ODD symptoms of children with ADHD were positively associated with higher caregiver parenting distress [40, 41]. Ross et al. also noted that mothers of children who received dual diagnoses of ADHD and ODD reported a higher level of stress rooted in the interactions with their children than did mothers of children with ADHD or ODD alone [42].

The present study is the first one to examine the relationship between caregivers’ acquiring knowledge of ADHD from social media and parenting stress. However, our study is not without limitations. First, our adoption of a cross-sectional research design limited our ability to confirm causal associations between the frequency of using sources of knowledge about ADHD and caregiver parenting stress and anxiety. Follow-up studies can provide evidence on the temporal relationship between the knowledge about ADHD acquired from various sources and caregiver parenting stress and anxiety. Second, because our data were collected solely through caregiver self-reports, our results may be affected by single-rater bias. Collecting data from multiple informants may reduce single-rater bias. Third, the present study recruited caregivers of children with ADHD exclusively from outpatient clinics; the findings therefore may not be generalizable to caregivers who do not seek care from child psychiatrists. Further study is needed to replicate the results of this study among caregivers of children with ADHD in community.

## Conclusion

In the present study, more than half of the caregivers of children with ADHD acquired knowledge about ADHD from social media, and acquiring knowledge about ADHD from social media was significantly associated with caregiver parenting stress. Moreover, acquiring knowledge about ADHD from the caregivers of other children was significantly associated with caregiver parenting stress and anxiety. Health-care professionals can alleviate such stress by using social media to disseminate accurate, reliable information about ADHD. Caregivers of children with ADHD must also exercise caution when appraising the quality of the information they receive about ADHD.

## Data Availability

No datasets were generated or analysed during the current study.

## References

[1] American Psychiatric Association (2013). Diagnostic and Statistical Manual of Mental Disorders, the Fifth Edition.

[2] Thomas R, Sanders S, Doust J, Beller E, Glasziou P (2015). Prevalence of attention-deficit/hyperactivity disorder: a systematic review and meta-analysis. Pediatrics.

[3] Rajaprakash M, Leppert ML (2022). Attention-deficit/hyperactivity disorder. Pediatr Rev.

[4] Perez Algorta G, Kragh CA, Arnold LE, Molina BSG, Hinshaw SP, Swanson JM (2018). Maternal ADHD symptoms, personality, and parenting stress: differences between mothers of children with ADHD and mothers of comparison children. J Atten Disord.

[5] Chronis AM, Lahey BB, Pelham WE, Kipp HL, Baumann BL, Lee SS (2003). Psychopathology and substance abuse in parents of young children with attention-deficit/hyperactivity disorder. J Am Acad Child Adolesc Psychiatry.

[6] Walls M, Cabral H, Feinberg E, Silverstein M (2018). Association between changes in caregiver depressive symptoms and child attention-deficit/hyperactivity disorder symptoms. J Dev Behav Pediatr.

[7] Gau SSF, Chang JPC (2013). Maternal parenting styles and mother-child relationship among adolescents with and without persistent attention-deficit/hyperactivity disorder. Res Dev Disabil.

[8] Wang LJ, Lee SY, Yuan SS, Yang CJ, Yang KC, Huang TS (2017). Prevalence rates of youths diagnosed with and medicated for ADHD in a nationwide survey in Taiwan from 2000 to 2011. Epidemiol Psychiatr Sci.

[9] Chang CC, Chen YM, Liu TL, Hsiao RC, Chou WJ, Yen CF (2020). Affiliate stigma and related factors in family caregivers of children with attention-deficit/hyperactivity disorder. Int J Environ Res Public Health.

[10] Chou WJ, Liu TL, Hsiao RC, Chen YM, Chang CC, Yen CF (2020). Application and perceived effectiveness of complementary and alternative intervention strategies for attention-deficit/hyperactivity disorder: relationships with affiliate stigma. Int J Environ Res Public Health.

[11] Janz NK, Becker MH (1984). The health belief model: a decade later. Health Educ Q.

[12] Haack LM, Meza J, Jiang Y, Araujo EJ, Pfiffner L (2018). Influences to ADHD problem recognition: mixed-method investigation and recommendations to reduce disparities for latino youth. Adm Policy Ment Health.

[13] Vander Stoep A, McCarty CA, Zhou C, Rockhill CM, Schoenfelder EN, Myers K (2017). The children’s attention-deficit hyperactivity disorder telemental health treatment study: caregiver outcomes. J Abnorm Child Psychol.

[14] Ahmed R, Borst JM, Yong CW, Aslani P (2014). Do parents of children with attention-deficit/hyperactivity disorder (ADHD) receive adequate information about the disorder and its treatments? A qualitative investigation. Patient Prefer Adherence.

[15] Bussing R, Zima BT, Mason DM, Meyer JM, White K, Garvan CW (2012). ADHD knowledge, perceptions, and information sources: perspectives from a community sample of adolescents and their parents. J Adolesc Health.

[16] Sciberras E, Iyer S, Efron D, Green J (2010). Information needs of parents of children with attention-deficit/hyperactivity disorder. Clin Pediatr.

[17] Rosenblum S, Yom-Tov E (2017). Seeking web-based information about attention deficit hyperactivity disorder: where, what, and when. J Med Internet Res.

[18] Li HH, Wang TT, Dong HY, Liu YQ, Jia FY (2022). Screening of ADHD symptoms in primary school students and investigation of parental awareness of ADHD and its influencing factors: a cross-sectional study. Front Psychol.

[19] Kivits J (2006). Informed patients and the internet: a mediated context for consultations with health professionals. J Health Psychol.

[20] Kisely S, Ong G, Takyar A (2003). A survey of the quality of web based information on the treatment of schizophrenia and attention deficit hyperactivity disorder. Aust N Z J Psychiatry.

[21] Sciutto MJ (2015). ADHD knowledge, misconceptions, and treatment acceptability. J Atten Disord.

[22] Cline RJ, Haynes KM (2001). Consumer health information seeking on the internet: the state of the art. Health Educ Res.

[23] Yeung A, Ng E, Abi-Jaoude E (2022). TikTok and attention-deficit/hyperactivity disorder: a cross-sectional study of social media content quality. Can J Psychiatry.

[24] Suarez-Lledo V, Alvarez-Galvez J (2021). Prevalence of health misinformation on social media: systematic review. J Med Internet Res.

[25] Julivia Murtani B, Wibowo JA, Liu CA, Rusady Goey M, Harsono K, Mardani AAP, Wiguna T (2020). Knowledge/understanding, perception and attitude towards attention-deficit/hyperactivity disorder (ADHD) among community members and healthcare professionals in Indonesia. Asian J Psychiatr.

[26] Park S, Lee Y, Lee ES, Kim CE (2018). Public recognition of attention-deficit hyperactivity disorder in Korea: correct identification, causes, treatments, and social distance. Asian J Psychiatr.

[27] Kaur G, Nitakumari K (2018). A descriptive study to assess the knowledge regarding ADHD among primary school teachers in selected schools of district Mohali with a view to develop informational booklet. Asian J Nurs Educ Res.

[28] Gau SSF, Shang CY, Liu SK, Lin CH, Swanson JM, Liu YC (2008). Psychometric properties of the Chinese version of the Swanson, Nolan, and Pelham, version IV scale–parent form. Int J Methods Psychiatr Res.

[29] Swanson JM, Kraemer HC, Hinshaw SP, Arnold LE, Conners CK, Abikoff HB (2001). Clinical relevance of the primary findings of the MTA: success rates based on severity of ADHD and ODD symptoms at the end of treatment. J Am Acad Child Adolesc Psychiatry.

[30] Weng Y (2011). Taiwanese version of the parenting stress index–short form.

[31] Abidin RR (2012). Parenting stress index, the Fourth edition.

[32] Che H-H, Lu M-L, Chen H-C, Chang S-W, Lee Y-J (2006). Validation of the Chinese Version of the Beck anxiety inventory. Formos J Med.

[33] Beck A, Norman E, Brown G, Steer RA (1988). An inventory for measuring clinical anxiety: psychometric properties. J Consult Clin Psychol.

[34] Kim H-Y (2013). Statistical notes for clinical researchers: assessing normal distribution (2) using skewness and kurtosis. Restor Dent Endod.

[35] Thapa P, Thapa A, Khadka N, Bhattarai R, Jha S, Khanal A (2018). YouTube lens to attention deficit hyperactivity disorder: a social media analysis. BMC Res Notes.

[36] Lopes LS, Valentini JP, Monteiro TH, Costacurta MCF, Soares LON, Telfar-Barnard L (2022). Problematic social media use and its relationship with depression or anxiety: a systematic review. Cyberpsychol Behav Soc Netw.

[37] Marino C, Mazzieri E, Caselli G, Vieno A, Spada MM (2018). Motives to use Facebook and problematic Facebook use in adolescents. J Behav Addict.

[38] Starke M, Möller A (2002). Parents’ needs for knowledge concerning the medical diagnosis of their children. J Child Health Care.

[39] Chou CP, Ho AH, Postiglione GA, Tan J (2007). Schooling in Taiwan. Going to School in East Asia.

[40] Wiener J, Biondic D, Grimbos T, Herbert M (2016). Parenting stress of parents of adolescents with attention-deficit hyperactivity disorder. J Abnorm Child Psychol.

[41] Harrison C, Sofronoff K (2002). ADHD and parental psychological distress: role of demographics, child behavioral characteristics, and parental cognitions. J Am Acad Child Adolesc Psychiatry.

[42] Ross CN, Blanc HM, McNeil CB, Eyberg SM, Hembree-Kigin TL (1998). Parenting stress in mothers of young children with oppositional defiant disorder and other severe behavior problems. Child Study J.

